# 4-Benzhydryl-1-cinnamylpiperazin-1-ium nitrate

**DOI:** 10.1107/S1600536808019958

**Published:** 2008-07-12

**Authors:** Zong-Ling Ru, Guo-Xi Wang

**Affiliations:** a Department of Chemical Engineering, Anyang Institute of Technology, Anyang 455000, People’s Republic of China

## Abstract

In the title compound, C_26_H_29_N_2_^+^·NO_3_^−^, the dihedral angle formed by the phenyl rings of the benzhydryl group is 66.18 (9)°. Crystal cohesion is enforced by cation–anion C—H⋯O and N—H⋯O hydrogen bonds.

## Related literature

For the use of amine derivatives in coordination chemisty, see: Manzur *et al.* (2007 ▶); Ismayilov *et al.* (2007 ▶); Austria *et al.* (2007 ▶).
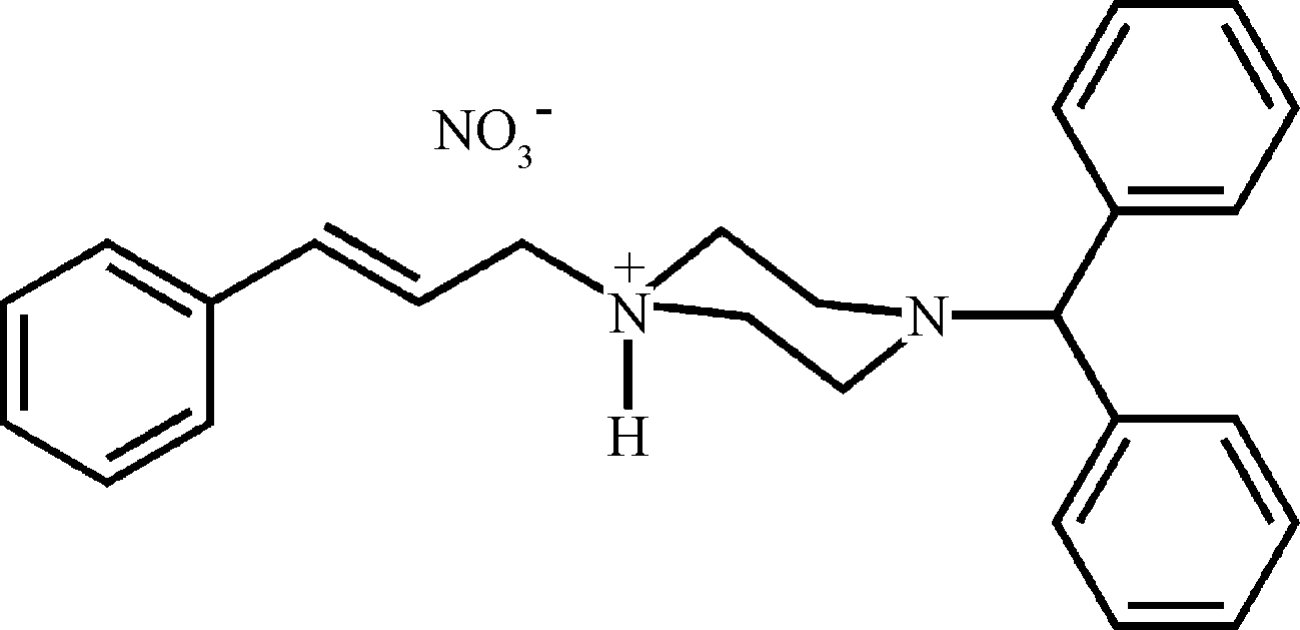


## Experimental

### 

#### Crystal data

C_26_H_29_N_2_^+^·NO_3_^−^
*M* *_r_* = 431.52
Monoclinic, 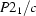

*a* = 18.6368 (17) Å
*b* = 10.8990 (10) Å
*c* = 12.0271 (10) Å
β = 107.397 (2)°
*V* = 2331.2 (4) Å^3^
*Z* = 4
Mo *K*α radiation
μ = 0.08 mm^−1^
*T* = 293 (2) K
0.27 × 0.18 × 0.15 mm

#### Data collection

Rigaku Mercury2 diffractometer
Absorption correction: multi-scan (*CrystalClear*; Rigaku, 2005 ▶) *T*_min_ = 0.978, *T*_max_ = 0.985
22444 measured reflections
5328 independent reflections
2413 reflections with *I* > 2σ(*I*)
*R*_int_ = 0.100

#### Refinement

*R*[*F* ^2^ > 2σ(*F* ^2^)] = 0.071
*wR*(*F* ^2^) = 0.178
*S* = 1.03
5328 reflections
289 parameters
H-atom parameters constrained
Δρ_max_ = 0.15 e Å^−3^
Δρ_min_ = −0.17 e Å^−3^

### 

Data collection: *CrystalClear* (Rigaku, 2005 ▶); cell refinement: *CrystalClear*; data reduction: *CrystalClear*; program(s) used to solve structure: *SHELXS97* (Sheldrick, 2008 ▶); program(s) used to refine structure: *SHELXL97* (Sheldrick, 2008 ▶); molecular graphics: *SHELXTL/PC* (Sheldrick, 2008 ▶); software used to prepare material for publication: *SHELXTL/PC*.

## Supplementary Material

Crystal structure: contains datablocks I, global. DOI: 10.1107/S1600536808019958/rz2224sup1.cif

Structure factors: contains datablocks I. DOI: 10.1107/S1600536808019958/rz2224Isup2.hkl

Additional supplementary materials:  crystallographic information; 3D view; checkCIF report

## Figures and Tables

**Table 1:** Hydrogen-bond geometry (Å, °)

*D*—H⋯*A*	*D*—H	H⋯*A*	*D*⋯*A*	*D*—H⋯*A*
N2—H2N⋯O3	0.90	2.02	2.862 (3)	156
N2—H2N⋯O1	0.90	2.31	3.101 (3)	146
C14—H14*A*⋯O3	0.97	2.53	3.263 (3)	132
C19—H19⋯O1	0.93	2.29	3.050 (4)	138

**Fig. 1.**
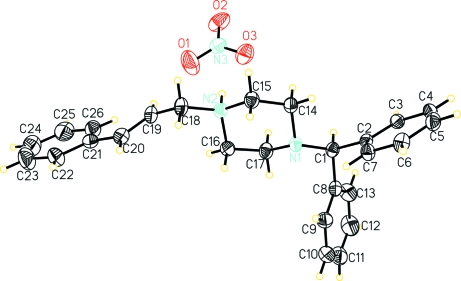
A view of the title compound with the atom numbering scheme. Displacement ellipsoids were drawn at the 30% probability level.

**Fig. 2.**
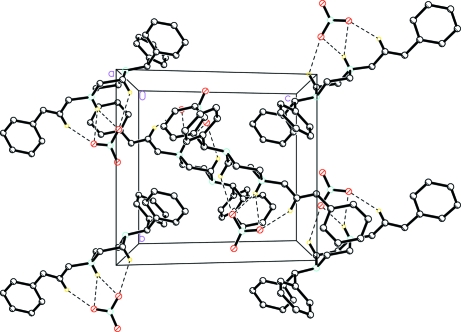
The crystal packing of the title compound viewed along the a axis. Hydrogen atoms not involved in hydrogen bonding (dashed lines) are omitted for clarity.

## supplementary crystallographic information

### Comment

In the past five years, we have focused on the chemistry of amine derivatives because of their multiple coordination modes as ligands to metal ions and for the construction of novel metal–organic frameworks (Manzur *et al.* 2007; Ismayilov *et al.* 2007; Austria *et al.* 2007). We report here the crystal structure of the title compound, 4-benzhydryl-1-cinnamylpiperazin-1-ium nitrate.

In the title compound (Fig. 1), the piperazine ring is protonated at the N2 atom and adopts the usual chair conformation. The phenyl rings of the benzhydryl group form a dihedral angle of 66.18 (9)°. The crystal packing is stabilized by C—H···O and N—H···O hydrogen bonds occurring between adjacent anions and cations (Table 1, Fig. 2).

### Experimental

4-Benzhydryl-1-cinnamylpiperazin-1-ium nitrate (3 mmol) was dissolved in ethanol (20 ml). The solvent was slowly evaporated in air affording colourless block-shaped crystals of the title compound suitable for X-ray analysis.

### Refinement

All H atoms were fixed geometrically and treated as riding with C—H = 0.93–0.97 Å, N—H = 0.93 Å, and with *U*_iso_(H) = 1.2*U*_eq_(C, N).

### Crystal data

**Table:**

C_26_H_29_N_2_^+^·NO_3_^–^	*F*_000_ = 920
*M**_r_* = 431.52	*D*_x_ = 1.230 Mg m^−^^3^
Monoclinic, *P*2_1_/*c*	Mo *K*α radiation λ = 0.71075 Å
Hall symbol: -P 2ybc	Cell parameters from 1178 reflections
*a* = 18.6368 (17) Å	θ = 2.3–24.4º
*b* = 10.8990 (10) Å	µ = 0.08 mm^−^^1^
*c* = 12.0271 (10) Å	*T* = 293 (2) K
β = 107.397 (2)º	Block, colourless
*V* = 2331.2 (4) Å^3^	0.27 × 0.18 × 0.15 mm
*Z* = 4	

### Data collection

**Table:**

Rigaku Mercury2 (2x2 bin mode) diffractometer	5328 independent reflections
Radiation source: fine-focus sealed tube	2413 reflections with *I* > 2σ(*I*)
Monochromator: graphite	*R*_int_ = 0.100
Detector resolution: 13.6612 pixels mm^-1^	θ_max_ = 27.5º
*T* = 293(2) K	θ_min_ = 3.0º
ω scans	*h* = −24→24
Absorption correction: multi-scan(CrystalClear; Rigaku, 2005)	*k* = −14→14
*T*_min_ = 0.978, *T*_max_ = 0.985	*l* = −15→15
22444 measured reflections	

### Refinement

**Table:**

Refinement on *F*^2^	Secondary atom site location: difference Fourier map
Least-squares matrix: full	Hydrogen site location: inferred from neighbouring sites
*R*[*F*^2^ > 2σ(*F*^2^)] = 0.071	H-atom parameters constrained
*wR*(*F*^2^) = 0.178	*w* = 1/[σ^2^(*F*_o_^2^) + (0.0622*P*)^2^ + 0.0525*P*] where *P* = (*F*_o_^2^ + 2*F*_c_^2^)/3
*S* = 1.04	(Δ/σ)_max_ < 0.001
5328 reflections	Δρ_max_ = 0.15 e Å^−^^3^
289 parameters	Δρ_min_ = −0.17 e Å^−^^3^
Primary atom site location: structure-invariant direct methods	Extinction correction: none

### Special details

**Table:**

Geometry. All e.s.d.'s (except the e.s.d. in the dihedral angle between two l.s. planes) are estimated using the full covariance matrix. The cell e.s.d.'s are taken into account individually in the estimation of e.s.d.'s in distances, angles and torsion angles; correlations between e.s.d.'s in cell parameters are only used when they are defined by crystal symmetry. An approximate (isotropic) treatment of cell e.s.d.'s is used for estimating e.s.d.'s involving l.s. planes.
Refinement. Refinement of *F*^2^ against ALL reflections. The weighted *R*-factor *wR* and goodness of fit *S* are based on *F*^2^, conventional *R*-factors *R* are based on *F*, with *F* set to zero for negative *F*^2^. The threshold expression of *F*^2^ > σ(*F*^2^) is used only for calculating *R*-factors(gt) *etc*. and is not relevant to the choice of reflections for refinement. *R*-factors based on *F*^2^ are statistically about twice as large as those based on *F*, and *R*- factors based on ALL data will be even larger.

### Fractional atomic coordinates and isotropic or equivalent isotropic displacement parameters (Å^2^)

**Table:**

	*x*	*y*	*z*	*U*_iso_*/*U*_eq_	
N1	0.31523 (11)	0.58781 (18)	0.45974 (16)	0.0422 (5)	
N2	0.30973 (11)	0.42385 (18)	0.26651 (16)	0.0460 (6)	
H2N	0.3154	0.3503	0.3020	0.055*	
C1	0.31968 (14)	0.6166 (2)	0.5816 (2)	0.0437 (6)	
H1	0.3244	0.5391	0.6244	0.052*	
C2	0.38828 (13)	0.6943 (2)	0.6397 (2)	0.0383 (6)	
C3	0.42973 (14)	0.6712 (2)	0.7539 (2)	0.0468 (7)	
H3	0.4148	0.6086	0.7947	0.056*	
C4	0.49302 (15)	0.7395 (3)	0.8085 (2)	0.0561 (8)	
H4	0.5204	0.7228	0.8853	0.067*	
C5	0.51513 (15)	0.8324 (3)	0.7483 (3)	0.0582 (8)	
H5	0.5581	0.8777	0.7840	0.070*	
C6	0.47373 (16)	0.8579 (3)	0.6358 (2)	0.0569 (7)	
H6	0.4883	0.9218	0.5960	0.068*	
C7	0.41097 (15)	0.7900 (2)	0.5814 (2)	0.0479 (7)	
H7	0.3834	0.8081	0.5050	0.057*	
C8	0.24792 (14)	0.6793 (2)	0.5869 (2)	0.0468 (7)	
C9	0.21408 (16)	0.7710 (3)	0.5104 (2)	0.0575 (8)	
H9	0.2360	0.7974	0.4546	0.069*	
C10	0.14769 (17)	0.8243 (3)	0.5158 (3)	0.0713 (9)	
H10	0.1256	0.8865	0.4641	0.086*	
C11	0.11438 (18)	0.7855 (4)	0.5974 (3)	0.0812 (11)	
H11	0.0689	0.8190	0.5993	0.097*	
C12	0.1490 (2)	0.6975 (4)	0.6754 (3)	0.0869 (11)	
H12	0.1279	0.6736	0.7331	0.104*	
C13	0.21470 (17)	0.6434 (3)	0.6702 (3)	0.0683 (9)	
H13	0.2369	0.5822	0.7232	0.082*	
C14	0.38274 (14)	0.5241 (2)	0.4509 (2)	0.0486 (7)	
H14A	0.3885	0.4472	0.4933	0.058*	
H14B	0.4267	0.5741	0.4862	0.058*	
C15	0.37754 (14)	0.4990 (2)	0.3257 (2)	0.0512 (7)	
H15A	0.3752	0.5763	0.2848	0.061*	
H15B	0.4224	0.4559	0.3224	0.061*	
C16	0.24141 (14)	0.4834 (2)	0.2819 (2)	0.0509 (7)	
H16A	0.1981	0.4309	0.2499	0.061*	
H16B	0.2324	0.5605	0.2396	0.061*	
C17	0.25099 (15)	0.5069 (2)	0.4092 (2)	0.0511 (7)	
H17A	0.2056	0.5443	0.4175	0.061*	
H17B	0.2586	0.4295	0.4510	0.061*	
C18	0.30351 (17)	0.4038 (3)	0.1408 (2)	0.0609 (8)	
H18A	0.2982	0.4827	0.1018	0.073*	
H18B	0.3497	0.3665	0.1355	0.073*	
C19	0.23928 (16)	0.3248 (3)	0.0793 (2)	0.0593 (8)	
H19	0.2316	0.2531	0.1161	0.071*	
C20	0.19265 (16)	0.3493 (3)	−0.0233 (2)	0.0628 (8)	
H20	0.1975	0.4255	−0.0550	0.075*	
C21	0.13313 (17)	0.2680 (3)	−0.0937 (3)	0.0636 (8)	
C22	0.10066 (18)	0.2921 (3)	−0.2118 (3)	0.0828 (11)	
H22	0.1139	0.3635	−0.2432	0.099*	
C23	0.0498 (2)	0.2140 (5)	−0.2832 (4)	0.1048 (14)	
H23	0.0300	0.2314	−0.3622	0.126*	
C24	0.0283 (2)	0.1098 (5)	−0.2373 (4)	0.1075 (15)	
H24	−0.0062	0.0564	−0.2854	0.129*	
C25	0.0578 (2)	0.0840 (4)	−0.1204 (4)	0.0945 (12)	
H25	0.0431	0.0137	−0.0893	0.113*	
C26	0.10931 (17)	0.1634 (3)	−0.0498 (3)	0.0749 (10)	
H26	0.1285	0.1462	0.0293	0.090*	
O1	0.29203 (17)	0.1418 (2)	0.2790 (2)	0.1197 (10)	
O2	0.35493 (15)	0.0310 (2)	0.4192 (2)	0.0982 (9)	
O3	0.36372 (13)	0.2268 (2)	0.42714 (19)	0.0849 (7)	
N3	0.33744 (15)	0.1316 (3)	0.3756 (2)	0.0622 (7)	

### Atomic displacement parameters (Å^2^)

**Table:**

	*U*^11^	*U*^22^	*U*^33^	*U*^12^	*U*^13^	*U*^23^
N1	0.0398 (13)	0.0489 (13)	0.0382 (12)	−0.0055 (10)	0.0118 (9)	−0.0046 (10)
N2	0.0558 (15)	0.0439 (13)	0.0385 (13)	−0.0008 (11)	0.0143 (10)	−0.0009 (10)
C1	0.0532 (17)	0.0407 (15)	0.0381 (15)	−0.0041 (12)	0.0151 (12)	−0.0006 (12)
C2	0.0403 (15)	0.0397 (15)	0.0359 (15)	−0.0006 (11)	0.0127 (11)	−0.0034 (11)
C3	0.0532 (18)	0.0455 (16)	0.0401 (16)	0.0042 (13)	0.0114 (13)	0.0018 (12)
C4	0.0506 (19)	0.070 (2)	0.0406 (17)	0.0096 (15)	0.0022 (13)	−0.0071 (15)
C5	0.0488 (18)	0.065 (2)	0.060 (2)	−0.0100 (15)	0.0149 (15)	−0.0179 (16)
C6	0.062 (2)	0.0584 (18)	0.0545 (19)	−0.0135 (15)	0.0245 (15)	−0.0067 (15)
C7	0.0544 (18)	0.0495 (16)	0.0373 (15)	−0.0059 (13)	0.0101 (12)	0.0002 (12)
C8	0.0466 (16)	0.0524 (17)	0.0412 (16)	−0.0062 (13)	0.0127 (13)	−0.0071 (13)
C9	0.0512 (19)	0.063 (2)	0.0585 (19)	0.0032 (15)	0.0174 (14)	−0.0024 (15)
C10	0.057 (2)	0.075 (2)	0.073 (2)	0.0049 (17)	0.0044 (17)	−0.0085 (17)
C11	0.045 (2)	0.118 (3)	0.078 (3)	0.005 (2)	0.0148 (19)	−0.025 (2)
C12	0.065 (2)	0.133 (3)	0.071 (2)	−0.007 (2)	0.0333 (19)	−0.008 (2)
C13	0.058 (2)	0.092 (2)	0.058 (2)	−0.0034 (17)	0.0229 (16)	0.0073 (17)
C14	0.0478 (18)	0.0520 (17)	0.0444 (17)	−0.0014 (13)	0.0112 (13)	−0.0062 (13)
C15	0.0487 (18)	0.0570 (18)	0.0492 (18)	−0.0074 (13)	0.0168 (13)	−0.0066 (13)
C16	0.0438 (17)	0.0532 (17)	0.0530 (18)	−0.0005 (13)	0.0102 (13)	−0.0064 (13)
C17	0.0460 (17)	0.0564 (18)	0.0512 (17)	−0.0112 (13)	0.0149 (12)	−0.0099 (14)
C18	0.074 (2)	0.074 (2)	0.0351 (17)	0.0019 (16)	0.0172 (14)	−0.0052 (14)
C19	0.070 (2)	0.064 (2)	0.0401 (17)	0.0021 (16)	0.0108 (15)	−0.0114 (14)
C20	0.069 (2)	0.064 (2)	0.055 (2)	0.0096 (16)	0.0179 (16)	−0.0050 (15)
C21	0.0481 (19)	0.080 (2)	0.060 (2)	0.0171 (16)	0.0123 (16)	−0.0114 (17)
C22	0.067 (2)	0.105 (3)	0.064 (2)	0.010 (2)	−0.0004 (17)	−0.006 (2)
C23	0.071 (3)	0.151 (4)	0.074 (3)	−0.005 (3)	−0.005 (2)	−0.021 (3)
C24	0.056 (3)	0.155 (4)	0.102 (4)	−0.007 (3)	0.008 (2)	−0.049 (3)
C25	0.060 (2)	0.115 (3)	0.110 (3)	−0.007 (2)	0.028 (2)	−0.024 (3)
C26	0.060 (2)	0.095 (3)	0.068 (2)	0.0024 (19)	0.0161 (17)	−0.017 (2)
O1	0.144 (2)	0.121 (2)	0.0685 (17)	−0.0040 (18)	−0.0074 (17)	0.0157 (16)
O2	0.163 (3)	0.0587 (14)	0.0891 (18)	0.0367 (15)	0.0623 (16)	0.0238 (13)
O3	0.105 (2)	0.0611 (15)	0.0901 (17)	−0.0110 (13)	0.0315 (14)	−0.0119 (13)
N3	0.0805 (19)	0.0556 (17)	0.0572 (18)	0.0114 (15)	0.0309 (14)	0.0090 (15)

### Geometric parameters (Å, °)

**Table:**

N1—C17	1.465 (3)	C14—C15	1.505 (3)
N1—C14	1.468 (3)	C14—H14A	0.9700
N1—C1	1.477 (3)	C14—H14B	0.9700
N2—C16	1.489 (3)	C15—H15A	0.9700
N2—C18	1.498 (3)	C15—H15B	0.9700
N2—C15	1.496 (3)	C16—C17	1.510 (3)
N2—H2N	0.9001	C16—H16A	0.9700
C1—C8	1.520 (3)	C16—H16B	0.9700
C1—C2	1.518 (3)	C17—H17A	0.9700
C1—H1	0.9800	C17—H17B	0.9700
C2—C3	1.382 (3)	C18—C19	1.480 (4)
C2—C7	1.391 (3)	C18—H18A	0.9700
C3—C4	1.383 (3)	C18—H18B	0.9700
C3—H3	0.9300	C19—C20	1.307 (3)
C4—C5	1.377 (4)	C19—H19	0.9300
C4—H4	0.9300	C20—C21	1.473 (4)
C5—C6	1.371 (4)	C20—H20	0.9300
C5—H5	0.9300	C21—C26	1.385 (4)
C6—C7	1.374 (3)	C21—C22	1.392 (4)
C6—H6	0.9300	C22—C23	1.369 (4)
C7—H7	0.9300	C22—H22	0.9300
C8—C9	1.377 (3)	C23—C24	1.374 (5)
C8—C13	1.382 (4)	C23—H23	0.9300
C9—C10	1.386 (4)	C24—C25	1.377 (5)
C9—H9	0.9300	C24—H24	0.9300
C10—C11	1.375 (4)	C25—C26	1.380 (4)
C10—H10	0.9300	C25—H25	0.9300
C11—C12	1.362 (4)	C26—H26	0.9300
C11—H11	0.9300	O1—N3	1.221 (3)
C12—C13	1.378 (4)	O2—N3	1.217 (3)
C12—H12	0.9300	O3—N3	1.233 (3)
C13—H13	0.9300		
			
C17—N1—C14	107.27 (19)	N1—C14—H14B	109.4
C17—N1—C1	109.66 (19)	C15—C14—H14B	109.4
C14—N1—C1	112.15 (18)	H14A—C14—H14B	108.0
C16—N2—C18	112.2 (2)	N2—C15—C14	111.6 (2)
C16—N2—C15	109.51 (19)	N2—C15—H15A	109.3
C18—N2—C15	111.1 (2)	C14—C15—H15A	109.3
C16—N2—H2N	108.2	N2—C15—H15B	109.3
C18—N2—H2N	108.2	C14—C15—H15B	109.3
C15—N2—H2N	107.4	H15A—C15—H15B	108.0
N1—C1—C8	110.39 (19)	N2—C16—C17	110.7 (2)
N1—C1—C2	111.41 (19)	N2—C16—H16A	109.5
C8—C1—C2	111.1 (2)	C17—C16—H16A	109.5
N1—C1—H1	107.9	N2—C16—H16B	109.5
C8—C1—H1	107.9	C17—C16—H16B	109.5
C2—C1—H1	107.9	H16A—C16—H16B	108.1
C3—C2—C7	118.3 (2)	N1—C17—C16	110.9 (2)
C3—C2—C1	120.0 (2)	N1—C17—H17A	109.5
C7—C2—C1	121.8 (2)	C16—C17—H17A	109.5
C2—C3—C4	121.2 (2)	N1—C17—H17B	109.5
C2—C3—H3	119.4	C16—C17—H17B	109.5
C4—C3—H3	119.4	H17A—C17—H17B	108.0
C5—C4—C3	119.5 (3)	C19—C18—N2	113.3 (2)
C5—C4—H4	120.3	C19—C18—H18A	108.9
C3—C4—H4	120.3	N2—C18—H18A	108.9
C6—C5—C4	120.0 (3)	C19—C18—H18B	108.9
C6—C5—H5	120.0	N2—C18—H18B	108.9
C4—C5—H5	120.0	H18A—C18—H18B	107.7
C5—C6—C7	120.6 (3)	C20—C19—C18	124.3 (3)
C5—C6—H6	119.7	C20—C19—H19	117.9
C7—C6—H6	119.7	C18—C19—H19	117.9
C6—C7—C2	120.5 (2)	C19—C20—C21	126.4 (3)
C6—C7—H7	119.8	C19—C20—H20	116.8
C2—C7—H7	119.8	C21—C20—H20	116.8
C9—C8—C13	118.3 (3)	C26—C21—C22	117.0 (3)
C9—C8—C1	122.1 (2)	C26—C21—C20	123.2 (3)
C13—C8—C1	119.6 (2)	C22—C21—C20	119.7 (3)
C8—C9—C10	120.6 (3)	C23—C22—C21	122.0 (4)
C8—C9—H9	119.7	C23—C22—H22	119.0
C10—C9—H9	119.7	C21—C22—H22	119.0
C11—C10—C9	120.3 (3)	C22—C23—C24	119.5 (4)
C11—C10—H10	119.8	C22—C23—H23	120.2
C9—C10—H10	119.8	C24—C23—H23	120.2
C12—C11—C10	119.1 (3)	C23—C24—C25	120.2 (4)
C12—C11—H11	120.4	C23—C24—H24	119.9
C10—C11—H11	120.4	C25—C24—H24	119.9
C11—C12—C13	120.9 (3)	C24—C25—C26	119.5 (4)
C11—C12—H12	119.5	C24—C25—H25	120.2
C13—C12—H12	119.5	C26—C25—H25	120.2
C12—C13—C8	120.6 (3)	C25—C26—C21	121.6 (3)
C12—C13—H13	119.7	C25—C26—H26	119.2
C8—C13—H13	119.7	C21—C26—H26	119.2
N1—C14—C15	111.0 (2)	O2—N3—O1	120.8 (3)
N1—C14—H14A	109.4	O2—N3—O3	121.8 (3)
C15—C14—H14A	109.4	O1—N3—O3	117.4 (3)
			
C17—N1—C1—C8	−59.4 (3)	C9—C8—C13—C12	0.6 (4)
C14—N1—C1—C8	−178.4 (2)	C1—C8—C13—C12	−178.9 (3)
C17—N1—C1—C2	176.61 (19)	C17—N1—C14—C15	61.1 (3)
C14—N1—C1—C2	57.6 (3)	C1—N1—C14—C15	−178.4 (2)
N1—C1—C2—C3	−139.5 (2)	C16—N2—C15—C14	53.2 (3)
C8—C1—C2—C3	96.9 (3)	C18—N2—C15—C14	177.7 (2)
N1—C1—C2—C7	40.9 (3)	N1—C14—C15—N2	−58.1 (3)
C8—C1—C2—C7	−82.6 (3)	C18—N2—C16—C17	−177.7 (2)
C7—C2—C3—C4	−1.2 (4)	C15—N2—C16—C17	−53.8 (3)
C1—C2—C3—C4	179.2 (2)	C14—N1—C17—C16	−62.3 (3)
C2—C3—C4—C5	0.1 (4)	C1—N1—C17—C16	175.7 (2)
C3—C4—C5—C6	1.2 (4)	N2—C16—C17—N1	60.1 (3)
C4—C5—C6—C7	−1.3 (4)	C16—N2—C18—C19	−59.8 (3)
C5—C6—C7—C2	0.2 (4)	C15—N2—C18—C19	177.2 (2)
C3—C2—C7—C6	1.1 (4)	N2—C18—C19—C20	134.3 (3)
C1—C2—C7—C6	−179.4 (2)	C18—C19—C20—C21	172.9 (3)
N1—C1—C8—C9	−43.9 (3)	C19—C20—C21—C26	13.1 (5)
C2—C1—C8—C9	80.2 (3)	C19—C20—C21—C22	−163.8 (3)
N1—C1—C8—C13	135.6 (2)	C26—C21—C22—C23	−2.7 (5)
C2—C1—C8—C13	−100.2 (3)	C20—C21—C22—C23	174.4 (3)
C13—C8—C9—C10	−1.1 (4)	C21—C22—C23—C24	1.6 (6)
C1—C8—C9—C10	178.5 (2)	C22—C23—C24—C25	0.0 (6)
C8—C9—C10—C11	−0.5 (5)	C23—C24—C25—C26	−0.3 (6)
C9—C10—C11—C12	2.5 (5)	C24—C25—C26—C21	−0.9 (5)
C10—C11—C12—C13	−2.9 (5)	C22—C21—C26—C25	2.3 (5)
C11—C12—C13—C8	1.4 (5)	C20—C21—C26—C25	−174.7 (3)

### Hydrogen-bond geometry (Å, °)

**Table:**

*D*—H···*A*	*D*—H	H···*A*	*D*···*A*	*D*—H···*A*
N2—H2N···O3	0.90	2.02	2.862 (3)	156
N2—H2N···O1	0.90	2.31	3.101 (3)	146
C14—H14A···O3	0.97	2.53	3.263 (3)	132
C19—H19···O1	0.93	2.29	3.050 (4)	138
